# A proposal for a primary screening tool: ‘Keep your waist circumference to less than half your height’

**DOI:** 10.1186/s12916-014-0207-1

**Published:** 2014-11-07

**Authors:** Margaret Ashwell, Sigrid Gibson

**Affiliations:** Ashwell Associates, Ashwell Street, Ashwell, Herts SG7 5PZ UK; Oxford Brookes University, Oxford, OX3 0BP UK; Sig-Nurture Ltd, Guildford, Surrey GU1 2TF UK

**Keywords:** Obesity, Waist-to-height ratio, Morbidity, Mortality

## Abstract

**Background:**

There is now overwhelming scientific evidence that central obesity, as opposed to total obesity assessed by body mass index (BMI), is associated with the most health risks and that the waist-to-height ratio (WHtR) is a simple proxy for this central fat distribution. This Opinion reviews the evidence for the use of WHtR to predict mortality and for its association with morbidity. A boundary value of WHtR of 0.5 has been proposed and become widely used. This translates into the simple screening message ‘Keep your waist to less than half your height’. Not only does this message appear to be suitable for all ethnic groups, it also works well with children.

**Discussion:**

Ignoring this simple message and continuing to use BMI as a sole indicator of risk would mean that 10% of the whole UK population, and more than 25% of the UK population who are judged to be normal weight using BMI, are misclassified and might not be alerted to the need to take care or to take action.

**Summary:**

Accepting that a boundary value whereby WHtR should be less than 0.5 not only lends itself to the simple message ‘Keep your waist to less than half your height’ but it also provides a very cheap primary screening method for increased health risks: A piece of string, measuring exactly half a person’s height should fit around that person’s waist.

## Background

### Use of BMI

The Body Mass Index (BMI) has served us well as a proxy for obesity for many years, but it has always been recognised that it does not differentiate between the muscular and the overweight, except at very high BMIs. But there is an even more important problem with BMI. Even in the overweight, it is only a proxy for total fat in the body and it does not distinguish between individuals with different types of fat distribution.

The first BMI chart, which displayed BMI as a function of weight (horizontal axis) and height (vertical axis) using contour lines for different values of BMI, first appeared in 1981 in John Garrow’s book *Treat Obesity Seriously* [1]. Since the early 1980s, the classic BMI chart has been used extensively to assess the severity of obesity. Healthy weight for height is usually defined as a BMI between 18.5 and 25 kg/m^2^, overweight as equal to or more than 25 and less than 30, and obesity as a BMI of equal to or more than 30 [2].

### Several indices to measure shape

Jean Vague [3] first pointed out in the 1940s and 1950s that people with a ‘central’ type of fat distribution (android shape) were at greater health risk than those whose fat was deposited ‘peripherally’ (gynoid shape). However, it has only been in the last few decades that there has been general agreement that health risks (predominantly cardiovascular disease (CVD) and diabetes) can be determined more by the relative distribution of the excess fat than by its total amount [4]. The use of imaging techniques such as computed tomography (CT) [5] and magnetic resonance imaging (MRI) [6] has subsequently indicated that the ‘unhealthy apple shape’ (Vague’s ‘android shape) is characterised by a preferential deposition of fat in the internal, visceral fat depots rather than in the external, subcutaneous fat depots which lead to the ‘healthy pear shape’ (Vague’s ‘gynoid’ shape).

Since this pioneering work, several indices to assess shape, such as waist circumference and the waist to hip ratio, have been proposed but there is not enough space here to present a complete history. Instead, the focus here will be on two indices which are currently in fashion. One such anthropometric index, the saggital abdominal diameter (SAD, could well be very accurate in predicting mortality risk [7,8] and another, a body shape index (ABSI), has been proposed as a way to quantify abdominal obesity [9]. However, SAD must be measured while the subject is prone and the calculation of ABSI is based on waist circumference relative to BMI and height and is very complicated. Both would not be suitable, or practical, for simple screening purposes.

### Proposal to use waist-to-height ratio in primary screening to assess shape

The ratio (R) of the waist circumference (W)-to-height (Ht) (WHtR) was originally proposed more or less simultaneously in Japan [10] and the UK [11-13] as a way of assessing shape and monitoring risk reduction. Both proposers suggested that WHtR values above 0.5 should indicate increased health risk.

We believe that a simple index such as WHtR is a good proxy for central obesity and has great practical advantages. The greater propensity for South Asians to develop diabetes at lower BMI than white Europeans has been recognised for some time leading to different BMI ranges being suggested for South Asians [14]. The use of WHtR circumvents such problems because the adjustment of waist circumference for height means that the same boundary values are suitable for both ethnic groups.

Here we summarise the evidence that WHtR is a good predictor for morbidity and mortality and then discuss the practical aspects.

### Morbidity

We recently conducted a systematic review of studies that have measured WHtR and BMI or waist circumference and looked at their relationship with metabolic risk factors, diabetes or CVD in adults or children [15]. Inclusion criteria were: human subjects, male, female or mixed, any age, adults or children, any ethnic group, novel studies, either prospective or cross-sectional design; WHtR and either BMI or waist circumference measured at least once; studies also had to have a mortality, a cardiometabolic disease endpoint or cardiometabolic risk outcome measure, and present the relationship between obesity and the disease endpoint or risk outcome.

Prospective and cross sectional studies (78 in all) showed odds ratios or correlations which were similar for all anthropometric indices, but tended to be higher for WHtR and waist circumference, than BMI. Further, WHtR and waist circumference tended to be significant predictors more often than BMI in all prospective analyses, which included nine studies with diabetes outcomes and fourteen studies with CVD outcomes. Thirteen cross-sectional analyses in children supported these predictions.

Analyses to determine the performance of each anthropometric index as a screening tool in adults (that is, assessing and comparing the diagnostic accuracy of different indices for a particular outcome), showed that WHtR was invariably a better tool than waist circumference or BMI. These specificity and sensitivity analyses were performed in more than 26 studies covering men and women in many ethnic groups including white European, South Asian, Afro Caribbean and Hispanic. The ages of subjects in these studies ranged from 18 to 100 years [15]. These data also confirmed that the cut-off (or boundary) value of WHtR 0.5 for increased risk is appropriate across age, gender and ethnic populations in adults.

Since our systematic review, many other investigators, especially those working in South Asia and South America, have also proposed that WHtR 0.5 be used for screening in many other populations. For example, in India [16,17], in Korea [18], in China [19], in Sri Lanka [20], in Spain [21] and in Chile [22]. The study by Cai *et al*. [19] analysed Chinese subjects in three age groups: 18 to 44 years, 45 to 59 years and 60 to 79 years. They showed that the discriminatory power of WHtR was better than BMI and waist circumference for identifying cardiometabolic risk in all age groups but noted that the discriminatory power of all indices was attenuated by age. Further research is needed in this area.

We have also proposed that the same boundary value of 0.5 and the same simple message of ‘Keep your waist to less than half your height’ would work well with children since their waist circumference increases as their height increases with age [23]. Recent research from cross sectional [24,25] and prospective studies [26,27] in children has supported this proposal.

### Mortality

The Health and Lifestyle Survey (HALS) is a longitudinal study of health and behaviour based on a representative random sample of the British population (England, Wales and Scotland). It was initiated in 1985 and now contains information on more than 20 years of follow-up data [28]. The dataset included more than 7,000 respondents from the age of 18. Nearly 2,000 deaths had been recorded up to 2005 and the relationship between BMI deciles (tenths) and mortality and WHtR deciles and all-cause mortality for men and women has recently been reported [29].

The conclusions were that WHtR is a more sensitive predictor of mortality than BMI for men and women (that is, based on a steeper mortality gradient across the deciles for WHtR). Use of regression analysis showed that the difference in slopes between mortality rate and BMI and WHtR was statistically different (*P* <0.01). Thus, the 20-year follow up data from HALS not only confirms the results from the 10-year data [13], but lends further support to the premise that WHtR is a superior predictor of mortality than BMI, particularly in the case of men.

### Simple practical screening with a shape chart based on WHtR

**Figure 1:**
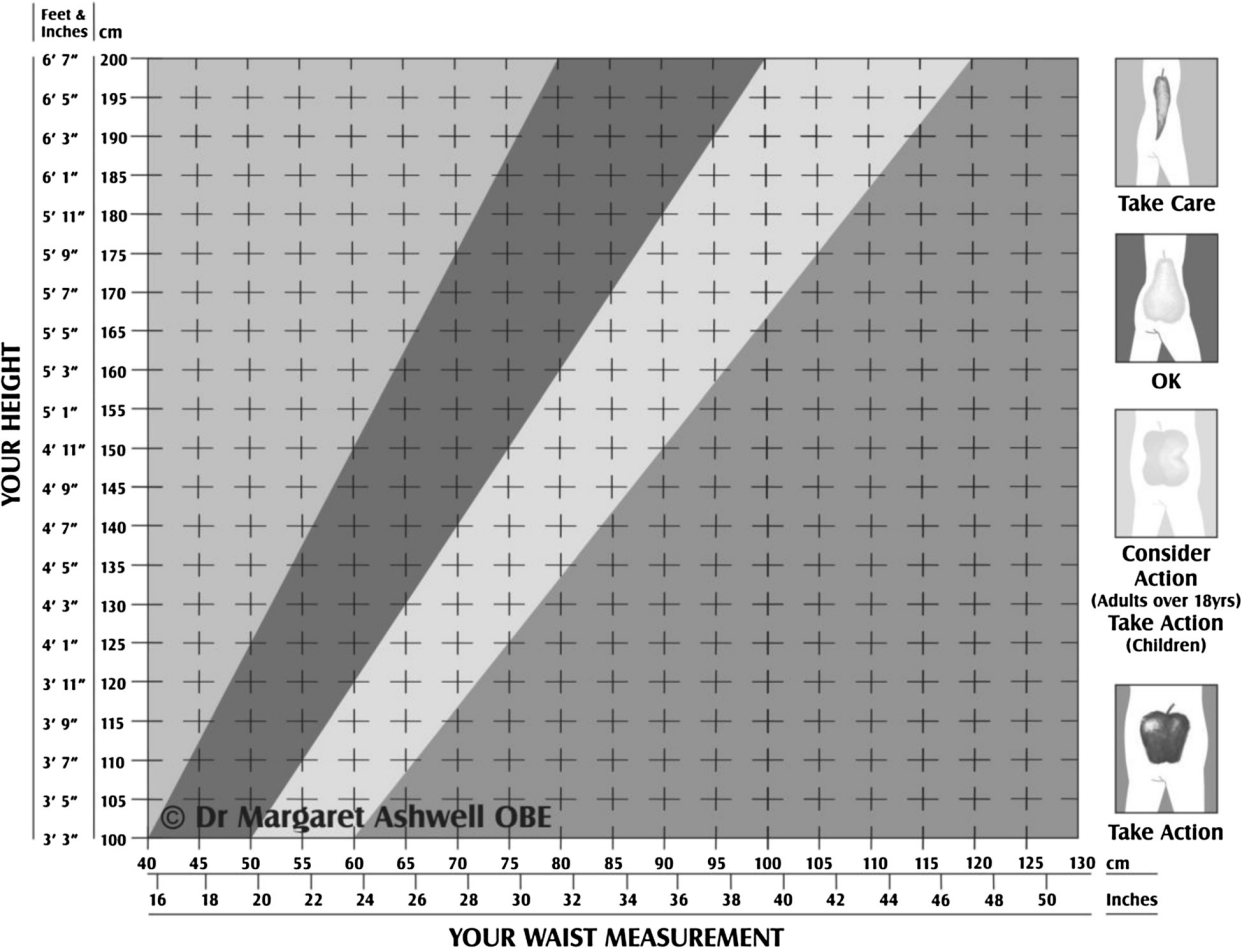
**The Ashwell (R) Shape Chart (copyright Dr Margaret Ashwell OBE).**

In the mid 1990s, one of us (MA) developed a chart (see Figure 1) based on WHtR (The Ashwell® Shape Chart) that allowed health professionals and/or their patients to match their waist circumference against their height- in inches or in centimetres- and to see into which category they fall [30]. Four regions based on boundary values for WHtR were designated. This is how they were described in terms of the ‘Action Steps’ for the patient:

- If your shape is in the ‘chilli’ region (WHtR less than 0.4), you should ‘Take Care’
- If your shape falls in the ‘pear’ region (WHtR between 0.4 and 0.5), you have a healthy ‘OK’ shape.
- If your shape falls in the ‘pear-apple’ region (WHtR between 0.5 and 0.6), you should ‘Consider Action’
- If your shape falls in the ‘apple’ region (WHtR above 0.6), your health is probably at risk. Why not talk with your health care provider, dietitian or practice nurse and ‘Take Action’?

Although the design of the chart has changed over the years, the boundary values for WHtR have not and there is now substantial evidence for them, especially the 0.5 boundary value. New actuarial data also give support for the 0.4 and 0.6 boundary values [31].

### What are the implications of continuing to use BMI and not using waist-to-height ratio for screening in the UK?

We have used recent data from four years of the UK National Diet and Nutrition Survey (NDNS) (2008 to 2012) to illustrate how the adult (19- to 64-year old) population is split using the traditional BMI boundary values and the proposed WHtR boundary value of 0.5. The NDNS sample is designed to be representative of free-living people 18-months old and older in the UK. Anthropometric measurements were obtained by trained personnel, and data on weight, height and waist circumference were available for 1,170 adults.

### How many people ‘at risk’ using BMI do not have high WHtR?

Table 1 shows that just under one in twenty of the population who are classed as overweight or obese by BMI (>25) have WHtR values which indicate they probably do not have central fat distribution. In other words, a proportion of adults are overweight/obese by BMI standards and yet are less likely to have associated health risks than those with similar BMIs and with central obesity. These people have been described elsewhere as metabolically healthy obese (MHO) and a systematic review has shown that the prevalence of MHO varies between 6% and 75% in different populations [32].

**Table 1:** **Adults misclassified by body mass index (BMI) revealed by waist-to-height ratio (WHtR)**

**BMI group**	**Numbers with WHtR equal or less than 0.5 (% in brackets)**	**Numbers with WHtR more than 0.5 (% in brackets)**	**Total in BMI category (% in brackets)**	**Percentage of each sex at risk by WHtR but missed by BMI screening**	**Percentage of each sex at risk by BMI but not at risk by WHtR**
**Men**					
Normal weight (BMI 18.5 to below 25)	103 (70%)	45 (30%)	148 (100%)	9% (45/505)	
Overweight and obese (BMI 25 and above)	17 (5%)	340 (95%)	357 (100%)		3% (17/505)
**Women**					
Normal weight (BMI 18.5 to below 25)	197 (74%)	70 (26%)	267 (100%)	11% (70/652)	
Overweight and obese (BMI 25 and above)	29 (8%)	356 (92%)	385 (100%)		4% (29/652)
**All adults**					
Normal weight (BMI 18.5 to below 25)	300 (72%)	115 (28%)	415 (100%)	10% (115/1157)	
Overweight and obese (BMI 25 and above)	46 (6%)	696 (94%)	752 (100%)		4% (46/1157)
**TOTAL** (excludes 13 adults with BMI <18.5)	346 (30%)	811 (70%)	1157 (100%)		

In Table 1, we have used recent data from four years of the UK National Diet and Nutrition Survey (NDNS) (2008 to 2012) to illustrate how the adult (19- to 64-year old) population is split using the traditional BMI boundary values and the proposed WHtR boundary value of 0.5. Cross-tabulation shows those with central fat distribution (WHtR >0.5) who would be ‘missed’ by BMI screening and those who are overweight/obese by BMI screening but do not have central fat distribution.

### How many people ‘at risk’ using WHtR will be missed by using BMI?

Of much greater concern is that Table 1 also shows that one in ten of the total population and more than a quarter of the ‘normal weight by BMI’ population have WHtR greater than 0.5 and are therefore ‘at risk’ because they have a central fat distribution. So continuing to use BMI would mean that a sizeable proportion of people would be ‘missed’ by screening on the basis of weight and height alone. The proportions of men and women at risk by WHtR, but missed by BMI, are 9% and 11%, respectively.

## Discussion

### Complexity of using BMI and waist circumference to assess risk

One of us (MA) has argued before in favour of keeping screening methods simple [33]. A very good example of the complexity of setting cut-off values for waist circumference and BMI was apparent recently in a study which compared the relationship between adiposity and prevalence of diabetes across ethnic groups in the UK Biobank cohort [34]. The proposed ethnic-specific obesity cut-offs that equate to those developed on white populations in terms of diabetes prevalence are shown in Table 2. For men and women, the BMI values for different ethnic groups that are equivalent to BMI 30 in white men and women range from 21.5 to 26. The Action levels for waist circumference in men and women in the different ethnic groups are also smaller than their white counterparts and show great variability. We have added our proposed WHtR boundary value of 0.5 to this table to illustrate the universality and simplicity of this boundary value.

**Table 2:** **Example to show simplicity of WHtR cut offs for different ethnic groups**

**Anthropometric measurements, by sex**	**White**	**South Asian (Pakistani)**	**South Asian (Indian)**	**Chinese**	**Black**
**Men**					
BMI (kg/m^2^)	30	21.5	22	26	26
Waist circumference (cm/in)	102/40	78/30.7	80/31.5	88/34.6	88/34.6
***Waist-to-height ratio (proposed by authors of this paper)***	***0.5***	***0.5***	***0.5***	***0.5***	***0.5***
**Women**					
BMI (kg/m^2^)	30	21.6	22.3	24	26
Waist circumference (cm/in)	88/34.6	68/26.7	70/27.5	74/29	79/31
***Waist-to-height ratio (proposed by authors of this Opinion)***	***0.5***	***0.5***	***0.5***	***0.5***	***0.5***

Table 2 shows the ethnic-specific BMI and waist circumference cut-offs that equate to those developed on white populations in terms of diabetes prevalence (proposed by [34]). Data are from the UK Biobank, which recruited 502,682 residents 40- to 69-years old. The table shows baseline data from the 490,288 participants from the four largest ethnic sub-groups. 96.1% were white, 2.0% were South Asian, 1.6% were black and 0.3% were Chinese. The waist-to-height ratio boundary values proposed by these authors (MA and SG) have been compared with these values to illustrate the universality and simplicity of this boundary value. These values are in bold italics to distinguish them from those generated from the Biobank data.

### Simplicity of using WHtR to assess risk

There is enough evidence now from all the ethnic groups portrayed in Table 2 to suggest that WHtR 0.5 makes a perfectly acceptable global cut-off value. For example, this value has been used in recent papers from India [16,17], in Korea [18], in China [19], in Sri Lanka [20], in Spain [21] and in Chile [22] and earlier studies from different ethnic groups were included in our meta-analysis [35].

In summary, we believe that a cut-off value of 0.5 for WHtR would be sufficient to indicate increased risk and that this value would be suitable for all ethnic groups. The inclusion of this simple value in Table 2 contrasts the simplicity of WHtR and the complexity of using cut-off values for BMI and waist circumference.

### Cheap primary screening using a piece of string to assess WHtR

The adoption of WHtR 0.5 as the most important boundary value for simple primary screening allows freedom from sophisticated and expensive measuring devices. The advice to ‘Keep your waist to less than half your height’ means that a piece of string can be cut to represent a person’s height and the same piece of string can be used folded in half to see if it fits around that person’s waist. If it does, ok. If it does not, this simple screening method has shown that further investigations of cardiometabolic risk factors should be made.

## Summary

The scientific evidence showing that WHtR is a better correlate of health risk than BMI is accumulating rapidly. These health risks include diabetes, hypertension, stroke, dyslipidemia and CVD. Translating science into policy always takes much longer. In this Opinion, we have presented new data to add to the case for using WHtR instead of BMI for primary screening purposes. We have shown that ten per cent of the whole population would be ‘missed’ if screening is only done on the basis of BMI. We have also pointed out the simplicity of measuring WHtR. If a tape measure is not available then a piece of string, which is folded so that it measures half a person’s height, can be used to show the ideal maximum waist circumference for health.

We make the plea that such a simple screening tool as a piece of string must be considered when the health risks from an obesity pandemic are acknowledged to be so great. Further, the problem is increasing rapidly in many countries which do not have access to more sophisticated measuring equipment.

Unwittingly, we have also come up with the answer to that age old question: How long is a piece of string? A piece of string could be cut to represent a person’s height and the same piece of string can be used (folded in half) to see if it fits around that person’s waist. If it does, ok. If it does not fit, then action to reduce the size of that person’s waist circumference is needed to reduce morbidity and mortality.
